# Improvement in Switching Characteristics and Bias Stability of Solution-Processed Zinc–Tin Oxide Thin Film Transistors via Simple Low-Pressure Thermal Annealing Treatment

**DOI:** 10.3390/nano13111722

**Published:** 2023-05-24

**Authors:** Junhao Feng, Sang-Hwa Jeon, Jaehoon Park, Sin-Hyung Lee, Jaewon Jang, In Man Kang, Do-Kyung Kim, Jin-Hyuk Bae

**Affiliations:** 1 School of Electronic and Electrical Engineering, Kyungpook National University, 80 Daehakro, Bukgu, Daegu 41566, Republic of Korea; suhuajunhao@knu.ac.kr (J.F.); masirong777@knu.ac.kr (S.-H.J.);; 2 Department of Electronic Engineering, Hallym University, Chuncheon 24252, Republic of Korea

**Keywords:** oxide semiconductor, solution process, thin film transistors, bias stability, low-pressure thermal annealing treatment

## Abstract

In this study, we used a low-pressure thermal annealing (LPTA) treatment to improve the switching characteristics and bias stability of zinc–tin oxide (ZTO) thin film transistors (TFTs). For this, we first fabricated the TFT and then applied the LPTA treatment at temperatures of 80 °C and 140 °C. The LPTA treatment reduced the number of defects in the bulk and interface of the ZTO TFTs. In addition, the changes in the water contact angle on the ZTO TFT surface indicated that the LPTA treatment reduced the surface defects. Hydrophobicity suppressed the off-current and instability under negative bias stress because of the limited absorption of moisture on the oxide surface. Moreover, the ratio of metal–oxygen bonds increased, while the ratio of oxygen–hydrogen bonds decreased. The reduced action of hydrogen as a shallow donor induced improvements in the on/off ratio (from 5.5 × 10^3^ to 1.1 × 10^7^) and subthreshold swing (8.63 to V·dec^−1^ and 0.73 V·dec^−1^), producing ZTO TFTs with excellent switching characteristics. In addition, device-to-device uniformity was significantly improved because of the reduced defects in the LPTA-treated ZTO TFTs.

## 1. Introduction

For the past several decades, amorphous oxide semiconductors (AOSs) have attracted attention as key unit devices for realizing next-generation display applications [1,2,3,4]. Compared to amorphous silicon, oxide semiconductors have overlapping neighboring s-orbitals because of the wide distribution of the spherical s-orbitals of their component metal cations. Therefore, the amorphous carriers in AOSs can be transported effectively in any direction, resulting in high mobility compared with amorphous silicon [5]. In addition, oxide semiconductors have high optical transparency owing to their wide bandgap [6]. In recent years, researchers have extensively studied indium-based oxide materials, such as indium oxide, indium gallium zinc oxide (IGZO), and indium zinc oxide. In particular, IGZO has gained much research attention, leading to its commercialization as a semiconductor layer in thin film transistors (TFTs) for active-matrix displays. However, indium and gallium are expensive rare metals, and hence they need to be replaced by other metal cations so as to achieve low-cost, next-generation display applications [7]. Thus, zinc–tin oxide (ZTO) has emerged as an amorphous rare metal–free semiconductor [8]. TFTs with ZTO semiconductors can achieve higher mobility than those with IGZO, but their excellent switching characteristics are difficult to obtain because of the absence of gallium, which acts as a carrier suppressor in oxide semiconductors [9]. Carrier suppressors such as gallium can easily bond with oxygen, thereby acting as an ingredient that inhibits defect formation, typically oxygen vacancy in oxide semiconductor systems [10].

Oxide TFTs have been fabricated using the vacuum process in industry. However, the vacuum process involves the formation of a vacuum environment inside a chamber, which is time consuming. In addition, the production of large-area TFTs is expensive and difficult. Thus, the solution process has received considerable attention as a next-generation manufacturing process for oxide TFTs because it can compensate for the shortcomings of the vacuum process [11,12]. Compared with vacuum processes, this process is relatively simple, fast, inexpensive, and easy to apply to large-area displays [13]. However, solution-processed oxide semiconductors have a higher density of chemical or physical defects than vacuum-processed semiconductors. Thus, they still require improvements in terms of device performance [14], stability [15], and device-to-device (D2D) uniformity [16]. In particular, the switching characteristics and bias stability should be improved so as to widen its applications in displays.

Nevertheless, it is difficult to achieve excellent switching characteristics and bias stability in ZTO semiconductors because of their high carrier concentration due to the absence of carrier suppressors, such as gallium [9]. Thus, more effort is needed to improve the switching characteristics and bias stability, as solution-processed ZTO TFTs have a high density of defects that originate from the solution process [17]. Moreover, the quality improvement of a semiconductor film through controlling physical and chemical defects can increase D2D uniformity in the active array, directly affecting the yield of the panel [18]. Therefore, there is a need for a facile approach that can improve switching characteristics, bias stability, and uniformity without compromising the simplicity of the solution process.

In this study, a low-pressure thermal annealing (LPTA) treatment was employed to fabricate solution-processed ZTO TFTs, with excellent switching property and bias stability. The effects of the LPTA treatment on the solution-processed ZTO TFTs were demonstrated by controlling the temperature during the treatment process. X-ray photoelectron spectroscopy (XPS), X-ray reflectivity (XRR), and field-emission transmission electron microscopy (FE-TEM) were used to investigate the physical and chemical properties of the ZTO TFTs.

## 2. Materials and Methods

**Figure 1:**
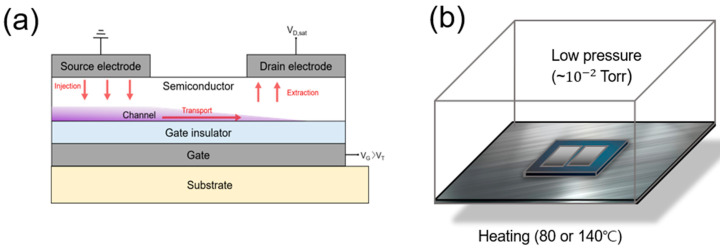
(**a**) Cross-sectional structure of a ZTO TFT. (**b**) Schematic of the LPTA treatment.

First, SnCl_2_, 2H_2_O, and ZnCl_2_ (Sigma Aldrich, St. Louis, MO, USA) were dissolved in 2-methoxyethanol(Sigma Aldrich, St. Louis, MO, USA) to prepare a solution containing 0.17 M ZTO. The Zn:Sn (Sigma Aldrich, St. Louis, MO, USA)molar ratio was 4:6 in the precursor solution. The solution was stirred for 1 h at room temperature to obtain a homogeneous, clear solution. SiO_2_/Si wafers were used as the gate dielectric and gate and were sonicated sequentially in acetone, isopropyl alcohol, and deionized water for 5 min each. This was followed by the removal of moisture from the substrate using N gas and subsequent heating to 150 °C on a hot plate. The synthesized ZTO solution was dropped onto a SiO_2_/Si substrate, which was then spin-coated at 4000 rpm for 15 s. After the coating stage, the pre-annealing stage was completed using a hot plate at 120 °C for 30 min. This was followed by a post-annealing stage at 500 °C for 30 min to densify and activate the oxide thin film. Then, 50 nm aluminum was deposited as the source-drain electrode using thermal evaporation. When considering previous studies, it was anticipated that the electrical characteristics of oxide TFTs could be enhanced by incorporating additional metals, particularly those with high work functions. Aluminum was adopted in this study because it has the advantages of low cost and easy formation as an electrode for TFT [19]. As shown in Figure 1a, the fabricated devices had a bottom-gate top-contact structure, and the channel width (W) and length (L) measured 1000 μm and 100μm, respectively. As depicted in Figure 1b, LPTA treatment was performed for 1 h at a temperature of 80 or 140 °C under a low-pressure condition of ~10^−2^ Torr using a vacuum dryer (OV-11, JeioTech, Daejeon, Republic of Korea). The ZTO thin films or TFTs without the LPTA treatment and with LPTA treatment at 80 °C and 140 °C were labeled as control group, LPTA-80, and LPTA-140, respectively. The LPTA treatment after the formation of source and drain electrodes might be desired due to the annealing process after the electrode deposition being able to decrease the contact resistance, thereby leading to the high performance of devices. The changes in the surface properties of the ZTO TFTs were confirmed by measuring the water contact angles using a drop shape analyzer (DSA100, Kruss, Hamburg, Germany). The chemical structure of the ZTO TFTs was analyzed via XPS (NEXSA, Thermo Fisher, Waltham, MA, USA). The thicknesses and densities of the TFTs were investigated using XRR (ATX-G, Rigaku, Tokyo, Japan) and FE-TEM (Titan G2 ChemiSTEM Cs Probe, FEI Company, Hillsboro, OR, USA). All devices were characterized using a probe station (MS Tech, T-4000A, Seoul, Republic of Korea) at room temperature and atmospheric pressure.

## 3. Results and Discussion

### 3.1. Physical Properties and Water Contact Angles of ZTO Thin Films

**Figure 2:**
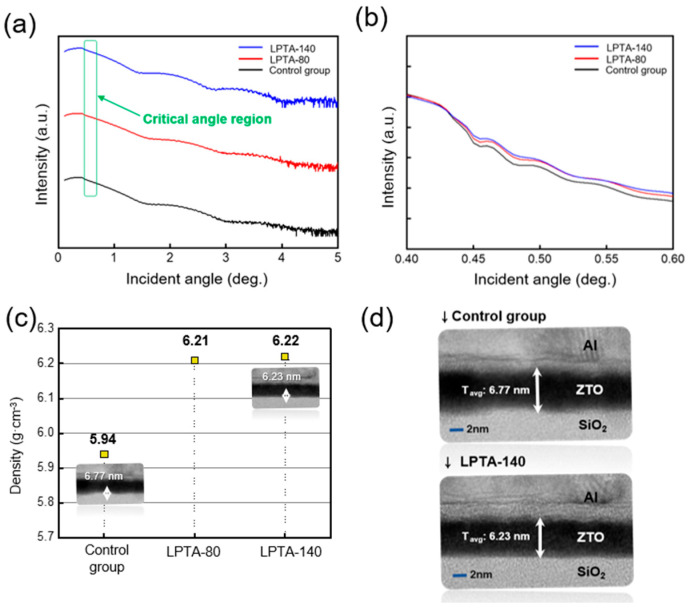
(**a**) XRR curves for the ZTO thin film with LPTA treatment at 80 °C (LPTA-80) and 140 °C (LPTA-140) and for that without LPTA treatment (control group). (**b**) Enlarged critical angle region of the control group, LPTA-80, and LPTA-140. (**c**) Film densities of the control group, LPTA-80, and LPTA-140. (**d**) FE-TEM image of the control group and LPTA-140.

The influence of the LPTA treatment on the physical characteristics of the ZTO thin films was investigated via XRR and TEM analysis. Note that all samples were fabricated and analyzed at the same time and in same environment to minimize the experimental error. Figure 2a,b present the obtained XRR curves and extracted film density results for the control group, LPTA-80, and LPTA-140, respectively. Figure 2b presents an enlarged critical angle region of Figure 2a to distinguish between small critical angle differences. The following Equation (1) [20] shows a proportional relationship between the film density (*ρ*) and the square of the critical angle (*θ_c_*).

(1) $$\theta_{c}^{2}=\left(\frac{e^{2}\lambda^{2}}{\pi mc^{2}}\right)\cdot \left(\frac{N_{A}Z}{A}\right)\rho$$

where *NA* denotes the Avogadro’s number, *λ* denotes the X-ray wavelength, *Z* denotes the average number of electrons per atom, and *A* denotes the average atomic mass. Figure 2c presents the film densities of the control group, LPTA-80, and LPTA-140 as 5.94 g·cm^−3^, 6.21 g·cm^−3^, and 6.22 g·cm^−3^, respectively. This finding indicates that LPTA treatment increases the density of thin films. In other words, the LPTA treatment can effectively densify TFTs by reducing defects and can enhance the overall film quality [21]. This observation was confirmed by the corresponding FE-TEM cross-sectional photographs of the control group and LPTA-140, presented in Figure 2d, as they revealed the average thicknesses of the control group and LPTA-140 to be 6.77 nm and 6.23 nm, respectively. Therefore, LPTA treatment contributed to the densification of ZTO thin films while suppressing physical defects such as pinholes and pore sites. This finding is consistent with the trend observed in film density via XRR analysis.

**Figure 3:**
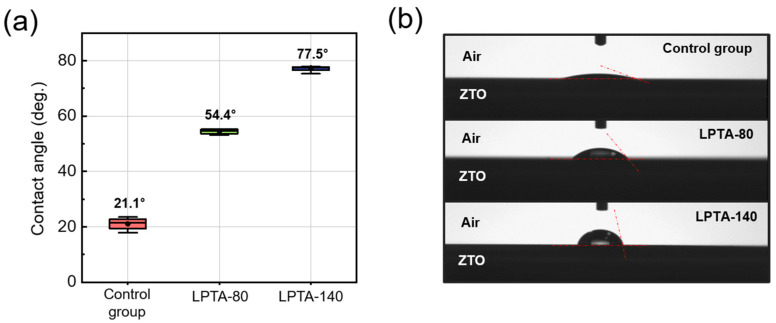
(**a**) Statistical data and average value and (**b**) photographs of the water contact angle on the surface of the control group, LPTA-80, and LPTA-140 (Red line represents the change of contact angle).

Water contact angles, defined as the angle between two interfaces on the intersection between the liquid–vapor interface and the solid interfaces of a droplet, were measured using the sessile drop method. Figure 3a shows the statistical data for the water contact angles of the control group, LPTA-80, and LPTA-140. Figure 3b presents the corresponding water droplet images on the ZTO thin film surface. The water contact angles were measured in eight devices for each condition. The average values of the measured water contact angles of the control group, LPTA-80, and LPTA-140 were 21.1°, 54.4°, and 77.5°, respectively.

The relationship between the water contact angle and the surface energy is described by Young’s equation [22]. An increased contact angle indicates the improved hydrophobicity of the surface [23]. Our findings showed that the contact angle of ZTO TFT increased with LPTA treatment and the increased treatment temperature. Thus, the surface of the control group exhibited strong hydrophilicity, while LPTA-140 had a hydrophobic surface. The hydrophilicity of the oxide surface may be proportional to the number of defects on the oxide surface [24]. Thus, the LPTA treatment can be interpreted to have improved the initial defective ZTO surface. In detail, the surface energy could cause the physisorption of ambient impurities, but all devices were exposed in the ambient environment at the same time. Thus, the surface energy difference, which is induced by LPTA treatment, might have originated from the reduction of surface defects. In addition, it decreased the hydrophilicity of the ZTO surface, affecting moisture adsorption and desorption on the oxide surface. H_2_O adsorbed on the back-channel surface in AOS TFTs acts as a donor and increases the concentration of the carrier [25]. The decreased hydrophilicity of the ZTO surface owing to the LPTA treatment impedes the absorption of moisture on such surfaces [23], thereby suppressing the off-current because of the back-channel passivation effect.

### 3.2. Chemical Properties of ZTO Thin Films

**Figure 4:**
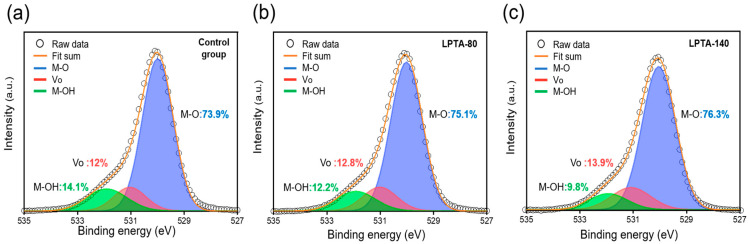
XPS O1s spectra of (**a**) control group, (**b**) LPTA-80, and (**c**) LPTA-140.

The effect of LPTA treatment on the chemical composition of ZTO TFTs was investigated using XPS analysis. Figure 4a–c display the XPS O1s spectra of the control group, LPTA-80, and LPTA-140, respectively. The XPS O1s spectra were deconvoluted using a Gaussian distribution with three binding energy peaks centered at ~529.8 eV, ~530.9 eV, and ~532.1 eV. The binding energy peak at ~529.8 eV corresponded to metal–oxygen (M-O) bonds, while that at ~530.9 eV corresponded to oxygen vacancy (V_o_). The highest binding energy peak at ~532.1 eV corresponded to weakly bound hydroxide groups (M-OH) [26]. The M-O bond ratios and the M-OH bond ratios for the control group, LPTA-80, and LPTA-140 were 73.9%, 75.1%, and 76.3%, and 14.1%, 12.2%, and 9.8%, respectively. Our findings showed that LPTA treatment reduced M-OH bonds in the thin film while also increasing the number of M-O bonds. In addition, the high-temperature environment during the LPTA treatment yielded a more significant change in the atomic bonding states of the ZTO semiconductors. This result suggests that the diffusion out of the weakly bonded hydrogen in the ZTO thin film structure from the low-pressure and thermal energy of the LPTA treatment decreased the M-OH bonds and increased M-O bonds [26]. Hydrogen inside the AOS structure exists as -OH bonds and donates free electrons as shallow donors [2,27,28]. The TFTs do not turn off in the case of extremely high free-electron density. Consequently, tuning the atomic bonding states and the diffusion out of hydrogen with the LPTA treatment reduced the carrier concentration and eventually suppressed the current flow in the off-state in ZTO TFTs. Moreover, the increased M-O bond ratio implies reduced defects within the thin film [29]. The Vo minutely changed the control group, LPTA-80, and LPTA-140 to 12%, 12.8%, and 13.9%, respectively. The increase in Vo through LPTA implies that the number of electrons generated by Vo increased. Although the change in Vo was interpreted using O1s spectrum in this study, it can also be interpreted using the M spectrum. The observed trend remains the same when Vo is interpreted through the M spectrum [30].

### 3.3. Effects of Low-Pressure Thermal Annealing on Electrical Characteristics in ZTO TFTs

**Figure 5:**
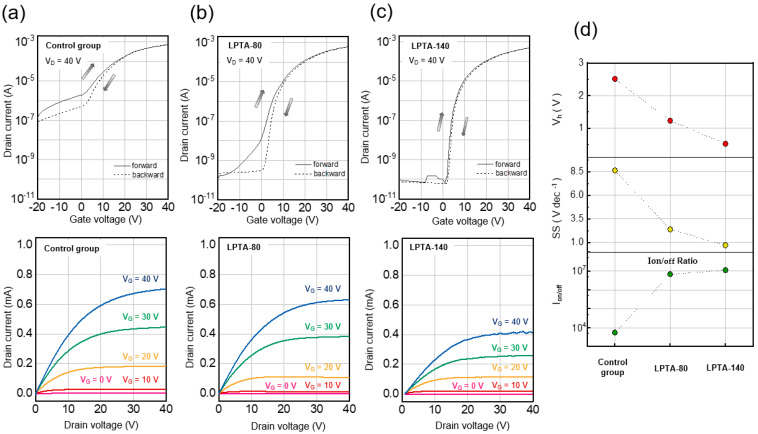
Transfer characteristics (**top**) and output characteristics (**bottom**) of (**a**) control group, (**b**) LPTA-80, and (**c**) LPTA-140. (**d**) Summary for Hysteresis (red), SS (yellow), and I_on/off_ ratio (green) of the control group, LPTA-80, and LPTA-140.

ZTO TFTs with different levels of low-pressure thermal annealing were fabricated to demonstrate the effect on their electrical characteristics and bias stability. Figure 5a–c present the transfer (top) and output characteristic (bottom) curves of the control group, LPTA-80, and LPTA-140, respectively. Transfer characteristics were measured by sweeping the gate voltage to −20 to 40 V while maintaining the drain voltage at 40 V. The output characteristics were measured by sweeping the drain voltage from 0 to 40 V and increasing the gate voltage from 0 to 40 V in increments of 10 V. All the devices were found to demonstrate saturation behavior at a drain voltage of 40 V. As shown in Figure 5, the transfer curves intuitively exhibited improved switching characteristics following LPTA treatment. In particular, the LPTA treatment exhibited a dramatic off-current reduction and a tremendous enhancement of the subthreshold swing (SS) characteristic. The control group displayed poor switching characteristics: the off-current, I_on/off_, and SS were found to be ~10^−7^ A, 5.5 × 10^3^, and 8.63 V·dec^−1^, respectively [31,32]. As depicted in Figure 5a, the off-current continued to increase before it was turned on, even when the device was turned off. The power consumption was high if the TFTs had a high current in the off-state. Meanwhile, as shown in Figure 5c, the off-current of LPTA-140 was approximately 10^−10^ A or less, and the I_on/off_ ratio was approximately 1.1 × 10^7^. Consequently, the LPTA treatment resulted in an off-current reduction of approximately 1000× and a tremendous improvement of 0.73 V·dec^−1^ in the SS characteristic. A moderate increase in the electron concentration improved only the on current while preserving the off current at a consistent level, resulting in an increase in the Ion/off. However, when the electron concentration becomes excessively high, it can lead to an increase in the off current, resulting in a decrease in I_on/off_. All devices showed clockwise hysteresis. The magnitude of hysteresis (*V*_h_) decreased from 2.5 V (control group) to 1.2 V (LPTA-80) and 0.52 V (LPTA-140), and was obtained by applying the following:

(2) $$V_{h}=V_{t,backward}-V_{t,forward}$$

where *V*_t,backward_ and *V*_t,forward_ denote the threshold voltage (*V*_t_) of the backward and forward sweeps, respectively. In addition, the LPTA treatment positively shifted *V*_t_ of the ZTO TFTs and reduced the on-current of the ZTO, as confirmed in Figure 5a–c. The change of *V*_t_ was attributed to the reduction in carrier concentration caused by the outward diffusion of hydrogen as the LPTA treatment. High carrier concentration leads to effective channel formation by facilitating the accumulation of electrons under a low vertical electric field. Figure 5d depicts the SS, I_on/off_ ratio, and hysteresis extracted from the transfer characteristics. Both the SS and clockwise hysteresis parameters were associated with the semiconductor-dielectric interface trap density (*N*_it_) [32,33,34], which is given as:

(3) $$N_{it}=\left[\frac{SSlog(e)}{kT/q}-1\right]\frac{C_{i}}{q}$$

where *k*, *T*, *q*, and *C*_i_ denote the Boltzmann constant, temperature, unit charge, and capacitance per unit area of the gate-insulator layer, respectively. The *N*_it_ values of the control group, LPTA-80, and LPTA-140 were determined to be 30.9 × 10^12^ cm^−2^·eV^−1^, 8.46 × 10^12^ cm^−2^·eV^−1^, and 2.42 × 10^12^ cm^−2^·eV^−1^, respectively [32]. Table 1 provides important information that can lead a better understanding of electrical characteristics in oxide TFTs with LPTA treatment and without LPTA treatment.

These findings indicate that the LPTA treatment reduced the trap states at the interface between the dielectric and semiconductor. The free carriers contributed to the channel accumulation, resulting in the negative shift of *V*_t_ and an increase in the on current. The increase in free carriers had a minimal impact on the off current, but a significant increase in free carriers would lead to an increase in the off current and poor switching characteristics due to the electron diffusion in negative Vg. The trapped carriers led to a positive shift in *V*_t_ and an increase in SS because the trapped electrons at trap sites cause a delay in the turn on of devices by delaying the channel accumulation. Considering the relevance of XPS results and electrical characteristics, the increase in M-O bonding that acts as charge transport path and the decrease in M-OH bonding that acts as trap site were dominantly affected electrical characteristics of TFTs rather than the slight increase in Vo. Recently, several methods have been reported to activate oxide semiconductors at low temperatures. If these methods are adopted, LPTA would become a more appealing choice because LPTA provides the advantage of low temperature processing, which is highly beneficial for TFT fabrication [11,37].

**Figure 6:**
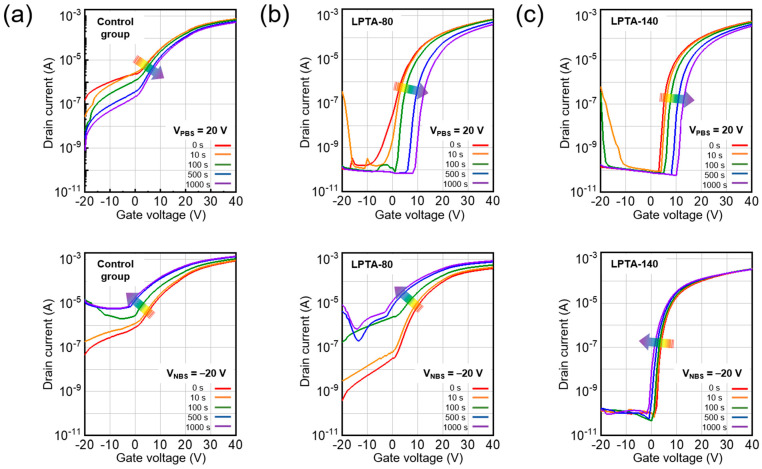
Transfer characteristics of (**a**) control group, (**b**) LPTA-80, and (**c**) LPTA-140 under PBS (**top**) and NBS (**bottom**).

Securing bias stability is an important factor for enhancing the practical use of TFTs because it plays an important role in determining the lifetime of a device. Figure 6a–c show the results of the positive bias stress (PBS; top) and negative bias stress (NBS; bottom) tests of the control group, LPTA-80, and LPTA-140, respectively. The gate bias was applied as 20 V and −20 V for PBS and NBS, respectively. As shown in Figure 6, the control group exhibited poor switching characteristics initially, and high instability under NBS and PBS. Meanwhile, LPTA-80 and LPTA-140 exhibited a low off-current even in PBS for 1000 s, and a marginal change in SSs in PBS. The findings suggest that charge trapping at the gate dielectric–semiconductor interface and injection into the gate dielectric rather than from the defect creation model resulted in the positive shift of *V*_t_ due to PBS because the SS related to the interface traps did not change as the stress time increased [38]. In addition, LPTA-140 showed a smaller positive shift in *V*_t_ compared with LPTA-80. The difference in the PBS-induced instability between the control group and LPTA-treated ZTO TFTs was attributed to electron trapping at the dielectric–semiconductor interface because the same thermally grown SiO_2_ dielectrics were used for ZTO TFTs in this study. As previously depicted by XRR and FE-TEM analyses, the ZTO semiconductor of LPTA-140 exhibited a high thin film density. This effective film densification contributed to the reduced trap density of the gate dielectric–semiconductor interface. The LPTA treatment also improved the stability of the device under NBS. In particular, the NBS stability of LPTA-140 dramatically improved compared with LPTA-80. Under NBS, LPTA-140 showed little change in the SS, off-current, and *V*_t_ shift, probably due to the decrease in the hydrophilicity of the ZTO surface from the LPTA treatment, as depicted in Figure 3a. In addition, the adsorption of H_2_O on the oxide surface became dominant under NBS, which increased the off-current and caused a negative shift in *V*_t_ [39]. In addition, the LPTA treatment could reduce the harmful effects of NBS as it impedes surface moisture adsorption. Hydrogen in ZTO TFTs could also affect the stability under NBS. As described in Figure 4, the migration of hydrogen-donated electrons in the thin film to the semiconductor-dielectric interface under negative bias formed positively charged hydrogen ions [40]. These positively charged hydrogen atoms may contribute to instability under NBS as donor-like traps.

**Figure 7:**
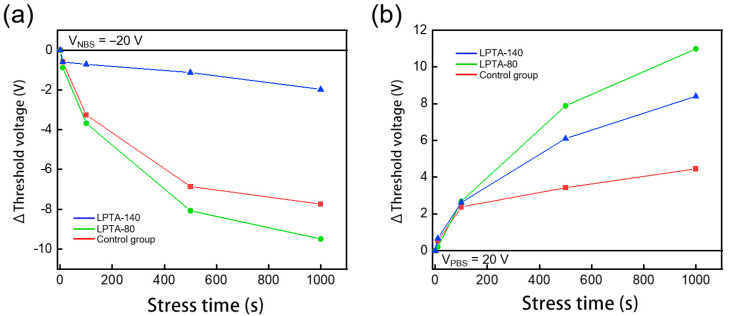
Stress time dependence of the *V*_t_ shift (Δ*V*_t_) of ZTO TFTs under (**a**) NBS and (**b**) PBS.

Figure 7 depicts the tendency for *V*_t_ shift in relation to the gate bias stress. The *V*_t_ shift of the control group displayed a relatively small variation in contrast to the trend of trap density discussed above. During NBS stress, both the control group and LPTA-80 showed a significant shift in *V*_t_, and the LPTA-80 exhibited a marginally larger variation. A weak level of LPTA also reduced the carrier concentration of the channel by removing moisture from the back-channel, but this could also be due to a failure to prevent moisture adsorption under NBS due to incomplete surface hydrophobicity. The *V*_t_ shift in the control group was the smallest under PBS. Channels appeared to form at low gate voltages despite the electric field screening effects by trapped electrons, probably because of an excessively high carrier concentration in the control group. As shown in Figure 6a, the drain current continued to rise even in the turn off region. Table 2 shows the tendency of the field-effect mobility (μ_FE_) according to gate bias stress. Overall, the control group had high μ_FE_ values. Moreover, the higher the concentration of the carrier in the channel, the higher the μ_FE_ in the percolation conduction model for oxide semiconductors [41].

### 3.4. Effects of Low-Pressure Thermal Annealing on D2D Uniformity in ZTO TFTs

**Figure 8:**
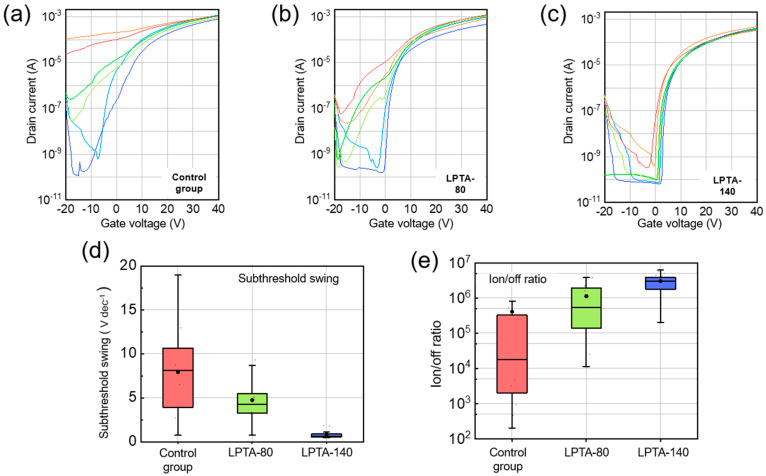
Transfer curves of (**a**) control group, (**b**) LPTA-80, and (**c**) LPTA-140. Here, 6 devices were selected out of 45 devices fabricated under the same conditions. Relatively good devices (sky-blue and blue lines), intermediate devices (yellow-green and green lines), and poor devices (orange and red lines) are shown. Statistical data for (**d**) SS and (**e**) I_on/off_ ratio of 45 devices for each condition.

Solution-processed oxide TFTs exhibited lower D2D uniformity than TFTs fabricated via vacuum processes [18]. In this study, 45 devices were manufactured for each condition (control group, LPTA-80, and LPTA-140) to confirm improvement in the D2D uniformity of the LPTA treatment. The SS and I_on/off_ ratio were extracted from the transfer curve for each device. All 45 devices for each condition were fabricated through four repeated experiments over four days. During this period, all the fabrication conditions, including spin-coating, pre-annealing, and post-annealing, were the same. As shown in Figure 8a–c, the transfer curves representing relatively good devices (sky-blue and blue lines), intermediate devices (yellow-green and green lines), and poor devices (orange and red lines) were selected from among the 45 devices for each condition. As shown in Figure 8a, some of the control groups rarely worked as transistors. In addition, the SS, I_on/off_ ratio, and turn-on voltage significantly differed for each device, indicating high nonuniformity in the control group. In contrast, LPTA-140 exhibited a small difference in switching characteristics compared with the control group and LPTA-80. Most LPTA-140 devices showed a constant turn-on voltage of approximately 0 V. Figure 8d,e present the statistical data of switching characteristics extracted from the 45 transfer curves for the control group, LPTA-80, and LPTA-140, respectively. The average SS was 7.93 V·dec^−1^, 4.73 V·dec^−1^, and 0.85 V·dec^−1^ for the control group, LPTA-80, and LPTA-140, respectively. Compared with the control group, almost all the LPTA-140 devices exhibited notably improved SSs owing to their low *N*_it_. The average log(Ion/off) values of the control group, LPTA-80, and LPTA-140 were 5.61, 6.05, and 6.48, respectively. As shown, LPTA-140 exhibited the highest average I_on/off_. The effect of improving the D2D uniformity was evaluated by determining the standard deviations of the SS and I_on/off_ ratio of the 45 devices for each condition. The standard deviations of the SS values were calculated to be 4.563 V·dec^−1^, 2.501 V·dec^−1^, and 0.485 V·dec^−1^ for the control group, LPTA-80, and LPTA-140, respectively. The standard deviations of the log(Ion/off) values were determined to be 1.252, 0.722, and 0.292 for the control group, LPTA-80, and LPTA-140, respectively. Moreover, the standard deviations of both SS and I_on/off_ tended to decrease, suggesting that a simple LPTA treatment could improve D2D uniformity by suppressing physical or chemical defects at the semiconductor–dielectric interface and ZTO surface, which could cause nonuniformity.

## 4. Conclusions

In this study, we significantly improved the switching characteristics of solution-processed indium-free oxide TFTs using a simple process. The results of the water contact angle measurements indicated that the LPTA treatment induced hydrophobicity of the ZTO surface, resulting in reduced surface defects and suppressed water absorption. This improved the off-state characteristics and stability under the NBS. In addition, the results of XRR, FE-TEM, and XPS analyses confirmed that the LPTA treatment improved the film density and chemical configuration of the ZTO thin film. The switching characteristics, bias stability, and D2D uniformity were significantly improved following LPTA-treatment-induced physical and chemical modifications of the ZTO semiconductor. We anticipate that LPTA treatment will be useful in improving the switching characteristics, bias stability, and nonuniformity of printable electronic devices. It also maintained the benefits of the solution process, such as low cost, high throughput, and simplicity. Moreover, the LPTA treatment might be effective for various oxide semiconductors and dielectric and conductor materials. As a future research direction, we plan to apply the LPTA treatment to oxide TFTs with low processing temperatures for high performance flexible electronics, because the processing temperature of LPTA is significantly low, making it a compatible treatment for flexible substrates.

## Figures and Tables

**Table 1:** Comparison with oxide TFTs.

	Ion/Off	Off-Current (A)	SS (V·dec^−1^)	*N*_it_ (cm^−2^·eV^−1^)	Reference
Control group	5.5 × 10^3^	~10^−7^	8.63	30.10 × 10^12^	This study
LPTA-80	6.7 × 10^6^	~10^−9^	2.40	8.46 × 10^12^	This study
LPTA-140	1.1 × 10^7^	~10^−10^	0.73	2.42 × 10^12^	This study
ZTO	5.2 × 10^3^	~10^−8^	1.52	-	[31]
ZTO	4.0 × 10^4^	~10^−8^	0.58	-	[35]
ZTO	3.0 × 10^7^	~10^−12^	0.39	1.20 × 10^12^	[36]
INO_X_	3.9 × 10^7^	~10^−12^	0.45	1.11 × 10^12^	[32]

**Table 2:** Summary of the field effect mobility (μ_FE_) of ZTO TFTs with different stress times under NBS and PBS.

μ_FE_ (cm^2^ V^−1^ S^−1^)	NBS			PBS		
Stress Time	Control Group	LPTA-80	LPTA-140	Control Group	LPTA-80	LPTA-140
0 s	5.1	3.36	1.99	3.39	2.89	2.87
10 s	5.37	3.59	1.99	4.10	2.88	2.89
100 s	5.64	3.91	2.09	4.35	3.23	3.00
500 s	6.15	4.21	2.26	3.85	3.39	3.07
1000 s	6.27	4.37	2.15	3.84	3.39	3.04

## Data Availability

Not applicable.

## References

[1] Fortunato E., Barquinha P. (2012). Martins, Oxide semiconductor thin-film transistors: A review of recent advances. Adv. Mater..

[2] Kamiya T., Hosono H. (2010). Material characteristics and applications of transparent amorphous oxide semiconductors. NPG Asia Mater..

[3] Nathan S., Lee S., Jeon J. (2014). Robertson, Amorphous oxide semiconductor TFTs for displays and imaging. IEEE/OSA J. Disp. Technol..

[4] Kamiya T., Nomura K., Hosono H. (2010). Present status of amorphous In-Ga-Zn-O thin-film transistors. Sci. Technol. Adv. Mater..

[5] Nomura K., Ohta H., Takagi A., Kamiya T., Hirano M., Hosono H. (2004). Room-temperature fabrication of transparent flexible thin-film transistors using amorphous oxide semiconductors. Nature.

[6] Shi J., Zhang J., Yang L., Qu M., Qi D.C., Zhang K.H.L. (2021). Wide Bandgap Oxide Semiconductors: From Materials Physics to Optoelectronic Devices. Adv. Mater..

[7] Fortunato E.M.C., Pereira L.M.N., Barquinha P.M.C., Rego A.M.B.D., Goņalves G., Vil A., Morante J.R., Martins R.F.P. (2008). High mobility indium free amorphous oxide thin film transistors. Appl. Phys. Lett..

[8] Fernandes C., Santa A., Santos Â., Bahubalindruni P., Deuermeier J., Martins R., Fortunato E., Barquinha P. (2018). A Sustainable Approach to Flexible Electronics with Zinc-Tin Oxide Thin-Film Transistors. Adv. Electron. Mater..

[9] Parthiban S., Kwon J.Y. (2014). Role of dopants as a carrier suppressor and strong oxygen binder in amorphous indium-oxide-based field effect transistor. J. Mater. Res..

[10] Kim S.J., Yoon S., Kim H.J. (2014). Review of solution-processed oxide thin-film transistors. Jpn. J. Appl. Phys..

[11] Park J.W., Kang B.H., Kim H.J. (2020). A Review of Low-Temperature Solution-Processed Metal Oxide Thin-Film Transistors for Flexible Electronics. Adv. Funct. Mater..

[12] Jeong S., Moon J. (2012). Low-temperature, solution-processed metal oxide thin film transistors. J. Mater. Chem..

[13] Glynn C., O’Dwyer C. (2017). Solution Processable Metal Oxide Thin Film Deposition and Material Growth for Electronic and Photonic Devices. Adv. Mater. Interfaces.

[14] Park J.S., Kim H., Kim I.D. (2014). Overview of electroceramic materials for oxide semiconductor thin film transistors. J. Electroceramics..

[15] Reed A., Stone C., Roh K., Song H.W., Wang X., Liu M., Lee S. (2020). The role of third cation doping on phase stability, carrier transport and carrier suppression in amorphous oxide semiconductors. J. Mater. Chem. C.

[16] Hwang Y.H., Seo J.S., Yun J.M., Park H.J., Yang S., Park S.H.K., Bae B.S. (2013). An “aqueous route” for the fabrication of low-temperature-processable oxide flexible transparent thin-film transistors on plastic substrates. NPG Asia Mater..

[17] Rim Y.S., Lim H.S., Kim H.J. (2013). Low-temperature metal-oxide thin-film transistors formed by directly photopatternable and combustible solution synthesis. ACS Appl. Mater. Interfaces.

[18] Kim K., Seo D., Kwon S., Jeon Y., Hwang Z., Wang J., Park S., Lee J., Jang I., Man X. (2022). Viable strategy to minimize trap states of patterned oxide thin films for both exceptional electrical performance and uniformity in sol–gel processed transistors. Chem. Eng. J..

[19] Tappertzhofen S., Waser R., Valov I. (2014). Impact of the Counter-Electrode Material on RedoxProcesses in Resistive Switching Memories. ChemElectroChem.

[20] Liu X., Xu H., Ning H., Lu K., Zhang H., Zhang X., Yao R., Fang Z., Lu X., Peng J. (2018). Induced Nano-Scale Self-Formed Metal-Oxide Interlayer in Amorphous Silicon Tin Oxide Thin Film Transistors. Sci. Rep..

[21] Rim Y.S., Jeong W.H., Kim D.L., Lim H.S., Kim K.M., Kim H.J. (2012). Simultaneous modification of pyrolysis and densification for low-temperature solution-processed flexible oxide thin-film transistors. J. Mater. Chem..

[22] Duca M.D., Plosceanu C.L., Pop T. (1998). Surface modifications of polyvinylidene fluoride (PVDF) under rf Ar plasma. Polym. Degrad. Stab..

[23] Ahmad D., Van Den Boogaert I., Miller J., Presswell R., Jouhara H. (2018). Hydrophilic and hydrophobic materials and their applications. Energy Sources Part A Recovery Util. Environ. Eff..

[24] Kim H.J., Tak Y.J., Park S.P., Na J.W., Kim Y.G., Hong S., Kim P.H., Kim G.T., Kim B.K., Kim H.J. (2017). The self-activated radical doping effects on the catalyzed surface of amorphous metal oxide films. Sci. Rep..

[25] Cho S.W., Kim D.E., Kim K.S., Jung S.H., Cho H.K. (2017). Towards environmentally stable solution-processed oxide thin-film transistors: A rare-metal-free oxide-based semiconductor/insulator heterostructure and chemically stable multi-stacking. J. Mater. Chem. C.

[26] Jeon J.K., Um J.G., Lee S., Jang J. (2017). Control of O-H bonds at a-IGZO/SiO2 interface by long time thermal annealing for highly stable oxide TFT. AIP Adv..

[27] Kamiya T., Hosono H. (2013). (Invited) Roles of Hydrogen in Amorphous Oxide Semiconductor. ECS Trans..

[28] Kamiya T., Nomura K., Hosono H. (2010). Subgap states, doping and defect formation energies in amorphous oxide semiconductor a-InGaZnO 4 studied by density functional theory. Phys. Status Solidi Appl. Mater. Sci..

[29] Tak Y.J., Park S.P., Jung T.S., Lee H., Kim W.G., Park J.W., Kim H.J. (2016). Reduction of activation temperature at 150 °C for IGZO films with improved electrical performance via UV-thermal treatment. J. Inf. Disp..

[30] Kyndiah A., Ablat A., Guyot-Reeb S., Schultz T., Zu F., Koch N., Amsalem P., Chiodini S., Alic T.Y., Topal Y. (2018). A Multifunctional Interlayer for Solution Processed High Performance Indium Oxide Transistors. Sci. Rep..

[31] Kim K.-Y., Kim T.G., Kim Y.H., Park J. (2015). Improved device performance of solution-processed zinc–tin–oxide thin film transistor effects using graphene/Al electrode. J. Phys. D Appl. Phys..

[32] Sabri M.M., Jung J., Yoon D.H., Yoon S., Tak Y.J., Kim H.J. (2015). Hydroxyl radical-assisted decomposition and oxidation in solution-processed indium oxide thin-film transistors. J. Mater. Chem. C.

[33] Jo J.W., Kim K.H., Kim J., Ban S.G., Kim Y.H., Park S.K. (2018). High-Mobility and Hysteresis-Free Flexible Oxide Thin-Film Transistors and Circuits by Using Bilayer Sol-Gel Gate Dielectrics. ACS Appl. Mater. Interfaces.

[34] Trinh T.T., Nguyen V.D., Ryu K., Jang K., Lee W., Baek S., Raja J., Yi J. (2011). Improvement in the performance of an InGaZnO thin-film transistor by controlling interface trap densities between the insulator and active layer. Semicond. Sci. Technol..

[35] Huang G., Duan L., Zhao Y., Dong G., Zhang D., Qiu Y. (2014). Enhanced mobility of solution-processed polycrystalline zinc tin oxide thin-film transistors via direct incorporation of water into precursor solution. Appl. Phys. Lett..

[36] Hsu C.-C., Chou C.-H., Chen Y.-T., Jhang W.-C. (2019). A study of solution-processed zinc-tin-oxide semiconductors for thin-film transistors. IEEE Trans. Electron Devices.

[37] Kim Y.-H., Heo J.-S., Kim T.-H., Park S., Yoon M.-H., Kim J., Oh M.S., Yi G.-R., Noh Y.-Y. (2012). Flexible metal-oxide devices made by room-temperature photochemical activation of sol–gel films. Nature.

[38] Jeong J.K. (2013). Photo-bias instability of metal oxide thin film transistors for advanced active matrix displays. J. Mater. Res..

[39] Xie H., Wu Q., Xu L., Zhang L., Liu G., Dong C. (2016). Nitrogen-doped amorphous oxide semiconductor thin film transistors with double-stacked channel layers. Appl. Surf. Sci..

[40] Um J.G., Mativenga M., Jang J. (2013). Mechanism of positive bias stress-assisted recovery in amorphous-indium-gallium-zinc-oxide thin-film transistors from negative bias under illumination stress. Appl. Phys. Lett..

[41] Takagi A., Nomura K., Ohta H., Yanagi H., Kamiya T., Hirano M., Hosono H. (2005). Carrier Transport and Electronic Structure in Amorphous Oxide Semiconductor, a-InGaZnO_4_. Thin Solid Film..

